# Risks of poor asthma outcome in 14,405 children and young people in London

**DOI:** 10.1038/s41533-020-00215-7

**Published:** 2021-01-29

**Authors:** Mark L. Levy

**Affiliations:** Locum General Practitioner, London, UK

**Keywords:** Asthma, Paediatrics

## Abstract

This is a 12-month retrospective data analysis (2018/19) of asthma risk factors in 350 North West London general practices. Fourteen thousand four hundred and five of the 482,029 (40% female) children and young people (CYP) had diagnosed asthma. Exacerbations are as follows: (i) 749 (5%) CYP had 797 hospital admissions; 32 (<1%) had 2–6; (ii) 910 (6%) had 1168 recorded asthma attacks; 170 (1%) had 2–12; (iii) 1485 (10%) had 2123 oral corticosteroid courses; 408 (3%) had 2–11. Excess short-acting bronchodilators were prescribed in over half of the CYP. Of the 10,077 (70%) CYP prescribed inhaled corticosteroid preventers, 7279 (72%) were issued with <4 ICS inhaler prescriptions during the year; these CYP accounted for 11% of the admission spells. In all, 30% of CYP had poor symptom control. At least 10% of the CYP having had recent attacks are at risk and dashboards such as those available in North West London could easily facilitate recognition of risk and optimisation of care.

## Introduction

The United Kingdom (UK) National Review of Asthma Deaths (NRAD) (2011–2014)^1^ identified a number of potentially preventable risk factors in those who died, many of which had been recognised previously^2,3^. While there has been localised implementation of some of the NRAD recommendations, to date only 1 of the 19 NRAD recommendations has been implemented nationally, i.e. the National Asthma and COPD Audit Programme, which is still to report on children. Sadly, despite the NRAD report highlighting ongoing preventable factors for asthma deaths, the UK’s poor record of childhood asthma care persists^4,5^ with many examples of preventable asthma attacks^6^ and preventable childhood asthma deaths^7–9^. In 2015/2016, Bedfordshire Clinical Commissioning Group (CCG) in England, UK performed a quality audit^4^ of 27,587 people of all ages diagnosed with asthma (prevalence 7%; range 4–12%) to identify the prevalence of some of the risk factors identified in the NRAD report. The results identified a wide variation in process of care and presence of risk factors, including excess short-acting bronchodilator reliever (short-acting beta-2-agonist (SABA) bronchodilators) and insufficient inhaled corticosteroid (ICS) controller prescriptions, failure to issue personal asthma action plans (PAAPs) or to perform annual reviews or to check inhaler technique.

In North West London (NWL), eight CCGs set up the Whole Systems Integrated Care (WSIC)^10,11^ data warehouse. WSIC provides an integrated care data set, updated twice monthly that includes information derived from acute, mental health, community, primary care and social care services. The WSIC data are available in de-identified format for research; however it is uniquely used to generate a dynamic up-to-date summary integrated care record from primary and secondary care, in the form of dashboards (called RADARs) accessible by authorised health and social care professionals involved in a patient’s treatment, thereby facilitating easy identification of people at clinical risk (see Supplementary Notes). The RADARs provide an opportunity to improve clinical decision-making and administration of National Health Service (NHS) patient care. The asthma Radar^12^ for example was designed to enable both generalists and asthma-trained clinicians to easily identify patients at risk of poor outcome by classifying patients with the most important risk factors displayed as visible ‘Red Flag’ markers derived from published evidence^1–3,13^. (Supplementary Fig. 1, with fictitious data for demonstration).

A de-identified version of the WSIC data set was utilised for the purpose of this study of children and young people (CYP) aged <19 years. UK general practitioners have utilised computerised data entry and prescription generating software since the early 1980s. Data have historically been coded in the UK either by utilising the hierarchical coding system devised by Dr. James Read or by entering free text; since 2019, the SnoMed system has been introduced for computerised coding of entries. Data entry by general practices (GPs) is further influenced by the UK Quality Outcomes Framework (QOF)^14^, which is used as a basis for determining GP payments. WSIC extracts data based on QOF-defined diagnosis, which in the case of asthma excludes any patients who have not had any asthma treatment prescribed in the previous 12 months.

A hypothesis was generated that asthma risk factors identified in the NRAD persist in the UK. This study aimed to investigate the current prevalence in NHS GPs of known risk factors for poor asthma outcome in CYP with doctor-diagnosed asthma meeting QOF criteria above in the 12 months from April 2018, utilising the WSIC data in NWL. These risk factors included excess prescription of SABAs, insufficient prescription of anti-inflammatory medication, asthma attacks/exacerbations, poorly controlled asthma and other published risk factors^2,3,13^.

## Results

Data were obtained for all CYP aged ≤18 years from all 350 GPs within NWL caring for 482,029 (49% female) CYP during the 12 months from April 2018. Twenty-one (6%) of the practices caring for 29,903 (6%) of the total CYP had no patients diagnosed with asthma in this age group. Of the CYP, 14,405 (3%), 40% female, were diagnosed and coded with asthma. Each CCG cared for a median of 60,253 CYP (interquartile range (IQR): 39,884–74,386, range 29,824–93,747) with a median of 2068 CYP patients (IQR: 1035–2428; range 756–2592) coded with asthma. Each of the 350 practices cared for a median of 1273 CYP (IQR: 786–1764, range 12–5626) with a median of 178 CYP (IQR: 93–265, range 3–350) in 329 of the practices diagnosed with asthma. The median prevalence of all CYP with diagnosed asthma in these 8 CCGs was 2.88% (IQR: 2.52–3.38%, range 2.49–3.52%) and within the practices was 3% (IQR: 2–4% range 0–10%) (see Table 1).

**Table 1:** Numbers of children and young people (CYP) diagnosed^a^ with asthma in 350 general practices in North West London in the 12 months from April 2018.

Gender	Details	0–<5 years	5–≤14 years	15–<19 years	Total
Female	Total CYP	74,057	115,559	45,195	234,811
	Asthma	404	3557	1708	5669
	Median % prevalence (Q1, Q3)	0.55 (0.4, 0.6)	3.08 (2.5, 3.6)	3.85 (3.26, 4.20)	2.41 (1.2, 2.7)
Male	Total CYP	77,981	121,416	47,809	247,206
	Asthma	629	5861	2246	8736
	Median % prevalence (Q1, Q3)	0.74 (0.6, 0.96)	4.62 (4.11, 5.61)	4.70 (4.20, 5.48)	3.53 (3.02, 4.03)

*Q1, Q3* 25th and 75th quartiles, CYP children and young people (aged ≤18 years).

^a^Coded in practice computer with an asthma diagnosis.

### Exacerbations

Exacerbations were defined as hospital admission for asthma attack or GP-coded entry for asthma attack or GP prescription for a short course of oral corticosteroids: during the 12 months: (i) 749 (5%) CYP were admitted to hospital for 797 episodes; 32 (<1%) of these CYP had between 2 and 6 admissions; (ii) 910 (6%) CYP were coded by the GPs as having had 1168 asthma attacks; 170 (1%) had between 2 and 12 asthma attacks; (iii) 1485 (10%) CYP had 2123 prescriptions for short courses of oral corticosteroids; 408 (3%) were prescribed between 2 and 11 courses (see Table 2). While each of the three defined exacerbation events were unique, it was not possible to determine the level to which these episodes overlapped.

**Table 2:** Age distribution of children and young people (CYP) diagnosed with asthma who had asthma exacerbations during 12 months (from April 2018).

Ages in years	Totals diagnosed	≥1 (%) Hospital^a^ admissions	≥2 (%) Hospital^a^ admissions	Coded^b^ with ≥1 (%) attacks	Coded^b^ with ≥2(%) attacks	Prescribed^c^ ≥1 (%) courses OCS	Prescribed^c^ ≥2 (%) courses OCS
0–≤5	1033	99 (10)	8 (1)	95 (9)	19 (2)	202 (20)	69 (7)
>5–≤14	9418	502 (5)	18 (0.2)	651 (7)	135 (1)	1012 (11)	271 (3)
>14–<19	3954	148 (4)	6 (0.4)	164 (4)	16 (0.5)	271 (7)	68 (2)
Total	14,405	749 (5)	32 (0.2)	910 (6)	170 (1)	1485 (10)	408 (3)

*CYP* children and young people ≤18 years, *OCS* oral corticosteroids.

^a^From hospital discharge data.

^b^Coded in General Practitioner records as having had an asthma attack.

^c^General Practitioner prescriptions for short courses of oral corticosteroids.

The hospital costs for the 797 admission spells for acute asthma during the 12 months were £1,058,117 and are detailed in Supplementary Table 1, with an average cost per annum for these admission spells of £73.45 per registered patient coded with asthma.

### Prescriptions

Of the 14,405 CYP diagnosed with asthma, 12,332 (86%) were prescribed a total of 35,427 SABA bronchodilator inhalers ranging between 1 and 22 per patient in the previous 12 months, with 5342 (43%), 1336 (11%) and 98 (1%) prescribed more than 3, 6 and 12 SABA inhalers in the 12 months; i.e. 43, 11 and 1% of these CYP were prescribed more than 12, 23 and 46 doses of SABA per week, respectively. The frequencies of and absolute numbers of SABA inhalers prescribed by the CCGs and variation between CCGs and practices is shown in Supplementary Table 2 and Supplementary Figs. 2 and 3. A multiple regression analysis of the SABA prescriptions in this population failed to demonstrate a statistically significant relationship between the number of SABA prescriptions and asthma exacerbations.

Of the 14,405 CYP coded with asthma, 4328 (30%) were not prescribed any ICSs either alone or in combination with a long-acting bronchodilator (long-acting beta-2-agonist) inhaler during the 12 months; i.e. 292 (28%), 2655 (28%) and 1381 (35%) of the CYP aged 0–≤5, >5–≤14 and ≥15–<19 years, respectively. Of the 10,077 CYP prescribed preventer inhalers, 7279 (72%) were issued with <4 ICS inhaler prescriptions during the year, and the numbers of ICS inhalers prescribed ranged from 1 to 24 per child per year. Overall, only 49% of the authorised repeat preventer medication prescriptions for these 14,405 children were collected from the doctor’s surgeries; range across the 8 CCGs was between 44 and 55%. Those children who collected <4 ICS preventer inhalers in the 12 months accounted for 11% of the admission spells in the 8 CCGs (see Table 3).

**Table 3:** Admission spells of those children and young people who were prescribed <4 inhaled preventer inhalers in 12 months.

CCGs	Total CYP with diagnosed asthma	Prescribed <4 ICS inhalers in 12 months, *n* (%)	Admission spells in those prescribed <4 ICS inhalers in 12 months, *n* (%)
1	2176	1096 (50)	153 (14)
2	756	336 (44)	45 (13)
3	2337	1287 (55)	103 (8)
4	1119	546 (49)	58 (11)
5	1960	1053 (54)	95 (9)
6	2458	1160 (47)	158 (14)
7	2592	1330 (51)	126 (10)
8	1007	471 (47)	59 (13)
Total	14,405	7279 (51)	797 (11)

*CCG* Clinical Commissioning Group, *CYP* children and young people ≤18 years, *ICS* inhaled corticosteroids.

### Other

There was considerable variation in recorded provision of PAAPs in the 329 practices (median 60%, IQR: 39–76%; Supplementary Table 3). It was not possible to establish the nature or quality of these plans nor of the training or qualifications of those providing them. On average, only 57% of the CYP patients with asthma in NWL had evidence in their practice record of having an annual asthma review (Supplementary Table 3).

Of the 13,372 CYP aged >5 years, inhaler technique was recorded in 8360 (63%), of whom the majority (93%) were recorded as having good technique. It was not possible to verify whether the inhaler technique was checked by someone trained to do so. Of those 2706 CYP (19%) who had their current asthma control measured during the study year, using the validated Asthma Control Test (ACT) score^15^, 815 (30%) had scores ≤19 (i.e. were poorly controlled when assessed).

## Discussion

Despite the poor record for childhood asthma outcomes in the UK and 5 years after the publication of the UK NRAD), at least 1485 (10%) of this population of CYP, having had recent attacks, and 30% with poor current control (ACT ≤19) remain at risk of poor outcomes (see Table 2), of whom at least 408 (3%) are at risk of potential life-threatening asthma attacks. See Box 2-2 GINA and table 14 SIGN/BTS guideline^2,3^. While numbers of deaths due to asthma in 0–24-year olds in England and Wales seems to have reduced^16^, the majority of deaths in this age group are preventable^1^. By identifying those at risk of poor outcomes and optimising their care, both unscheduled care for asthma and avoidable deaths can be reduced.

Nearly 6 years after the NRAD report^1^ and subsequent high profile UK child death inquest reports^7–9^, 19% of the NWL CYP were prescribed excess SABA inhalers known to be potentially harmful when used regularly^17,18^, with at least 1% potentially at risk of life-threatening attacks due to excess SABA prescription and at least 3% having had two or more recent attacks^1–3,19,20^. That 49% of the 14,405 CYP coded with a diagnosis of asthma were prescribed <4 ICS inhalers in the 12 months and that a third of those who had an asthma control test in the year were poorly controlled is of concern.

While data for all of the practices in NWL was extracted, 21 (6%) had no CYP diagnosed with asthma. It was not possible to establish whether any CYP in these practices were being treated with asthma medication without a diagnosis; a problem noted in a UK study of >600 children being treated for asthma, 25% of whom were not coded on the practice asthma registers^21^. As with any database study, the quality of WSIC data extracted depends upon that entered by the clinicians. The laboratory data are imported directly from laboratory sources, prescribing data are almost 100% accurate, apart for the few that may be issued manually when visiting patients at home and not updated on the practice computer systems. This study utilised real-life data recorded in primary and secondary care. Compared with England and Wales, asthma in CYP is underdiagnosed in NWL. The prevalence ranged from 2.53 to 3.52%, which is considerably lower than figures published by Bloom et al.^22^, where the average UK prevalence in children aged <5, 5–11 and 12–17 years was 1.6, 6.83 and 7.96%, respectively. This low prevalence may be partly explained by the way GP-diagnosed asthma is classified in the UK QOF used for payment of GPs^23^ and due to possible underdiagnosis for example in the 21 practices who had no CYP cases coded with an asthma diagnosis. The WSIC database extracts data for people coded with chronic disease according to the QOF definitions, i.e. asthma is confirmed if the record is coded with asthma and there has been a prescription in the previous 12 months. It was not possible (due to budget) to extract data for patients treated with asthma medication who were not coded with an asthma diagnosis.

The definition, for the purpose of this study, for asthma exacerbations included hospital admissions and/or GP-coded episodes of asthma attacks and/or GP prescriptions for short courses of oral corticosteroids. While prescriptions for OCS are used as an outcome and/or a marker for an attack in most published studies investigating asthma medications and care^24^, these may not always be prescribed appropriately^25^. In this population of CYP, at least 5% had suffered from severe asthma attacks (i.e. had been admitted to hospital); however, it is unclear how many of the 1485 children prescribed 2123 short courses of oral prednisolone had severe attacks without being admitted to hospital. A common problem in the UK is that severity of asthma attacks is not often assessed objectively^1^, as recommended in the current SIGN/BTS guideline, table 17^3^. While there is uncertainty of the level of overlap in those classified with exacerbations, it is clear from this data that not all asthma exacerbations were coded as such by the general practitioners, making it difficult to identify those in need of a post exacerbation review as recommended in NICE^26^ and the SIGN/BTS^3^ guidelines.

Utilising prescriptions alone as a measure of quality is clearly insufficient; ideally this needs to be combined with verified assessment of inhaler technique and adherence to prescribing advice, which cannot be done when retrospectively analysing computerised records. The use of numbers of prescriptions for SABA as a risk factor for poor asthma outcome^1,19^ could be challenged in the UK where some doctors routinely prescribe salbutamol regularly, and furthermore it has become common practice in paediatrics for example to prescribe unlicenced, non-evidence based ‘weaning doses’ comprising 10 puffs of salbutamol SABA 4–6 hourly for up to 5 days post attacks^27^. However, this practice is potentially unsafe and there are increasing levels of evidence that >3 SABA prescriptions a year are associated with increased attacks, hospital admissions and deaths^17,18,28^. Four or more prescribed ICS inhalers in 12 months was chosen as a convenient measure of the minimum annual number of ICS inhalers required (e.g. beclomethasone inhalers, 200 doses at 1 puff twice a day). Therefore, the 11–14% of CYP prescribed <4 ICS (see Table 3) admitted for asthma attacks may be an underestimate of those collecting insufficient ICS (i.e. if 12 a year should have been collected e.g. where one device a month was required according to the dose prescribed) or not adhering to their prescribed dosages. The study identified that 30% of these CYP were not prescribed any preventer inhalers during the 12 months; however, it was not possible to determine what proportion of these children accounted for the admission spells. However, one Australian study found that 17–60% of CYP with varying levels of asthma severity who had not previously been prescribed ICSs accounted for hospital admissions^29^. Therefore, it is likely that this study has underestimated the consequences of under-prescribing ICSs in CYP with asthma.

Recorded provision of an asthma personal action plan and annual asthma reviews (including checking inhaler technique) are two of the criteria for payment of practices under the QOF, and while the provision of plans and reviews in this sample of practices is considerably higher than in previously published UK data^1^, the type or quality of provision of these plans or reviews cannot, from this data, be verified, nor whether these items were simply recorded as completed. Furthermore, as many health care professionals are unable to correctly use and therefore assess inhaler technique^30^, the quality of the inhaler technique assessments detailed within the database cannot be verified.

Under-prescription of preventer medication^1,31,32^ and over prescribed SABA^1,2,17–20^ are well-recognised risk factors for poor outcomes (including death), and in the case of children, failure of parents to collect medication^6^ or attend for reviews^1,9^ are indications of possible safeguarding issues^9^, which should be recognised for further investigation by clinicians.

That 93% of CYP whose inhaler technique was tested was recorded as ‘good’ was a very surprising finding given the evidence in the literature that most people cannot use their inhalers correctly^33^. Furthermore, neither the method used nor the level of training of those health care professionals who recorded the inhaler technique in these CYP could be ascertained.

The costs of 797 admission spells reported in this study relates only to those patients coded with an asthma diagnosis; however, the numbers of CYP admitted who were being treated with asthma medication and not coded with asthma could not be ascertained. Lo et al.^21,34^ showed that about 25% of children treated were not coded with asthma by their GPs; therefore, the data probably underestimate the true prevalence of asthma and cost of admissions in NWL.

The data in this population of CYP in NWL demonstrate that excess prescribing of SABAs and under-prescription of preventer medication persists despite evidence that this is unsafe practice. In those prescribed insufficient ICS, there was an association with increased numbers of severe asthma attacks requiring hospitalisation in this childhood population. Research has demonstrated well-known risks of poor outcomes^35^, including death due to overuse of regular SABA and benefits of even small doses of ICS^31,36^. With such persuasive evidence available, it is time for all involved in commissioning and delivering asthma care to take action to reduce morbidity and save lives. A real-time national system for collecting and collating asthma data immediately available for clinicians, such as the unique WSIC Radar dashboards in NWL (see Supplementary Notes and Supplementary Fig. 1) could facilitate easy identification of those at risk. Asthma care must be provided by appropriately trained individuals who understand that an attack signifies a failure of management requiring identification and optimisation of modifiable risk factors. This approach has resulted in improved quality of life, reduced unscheduled care, reduced deaths and substantial financial savings in Finland^37–39^.

## Methods

This was a retrospective GP and hospital data analysis over 12 months from April 2018 of the NWL WSIC data warehouse^10,12^, which was set up and funded by a NWL Collaboration of eight CCGs. Using unique NHS identification number linkage, computerised data are extracted twice monthly for 2.2 million patients from >400 provider organisations, including 350 GP practices, 2 mental health and community trusts and all acute providers. Patients and practices have the option of withholding consent to share their data.

A fully de-identified version of the WSIC data delivered by the Imperial Health Care Partnership with WSIC called Discover-Now^40^ is available for research. In addition, uniquely, the data are used to facilitate real-time clinical and administrative decision-making. This is accessible to authorised health and social care professionals involved in patients’ treatment through a number of data dashboards (called RADARs), including an asthma RADAR^12^ (Supplementary Fig. 1). This combination of dynamic up-to-date clinical data used both for research and clinical care is the only such system in the UK and possibly the world.

The WSIC data were used to describe the NWL prevalence of asthma risk factors in CYP^1^, such as excess prescriptions for SABA relievers and insufficient preventer medication and unscheduled care for exacerbations. For the purpose of this study that utilised real-life data entered by general practitioners and their staff, exacerbations were defined in three ways, i.e. those people: (i) admitted to hospital (derived from actual hospital discharge data coded as acute asthma attacks) and/or (ii) coded by the GPs on their computers as having had asthma attacks and/or (iii) prescribed short courses of oral corticosteroids by the GPs or their staff. The Discover-NOW senior data analyst extracted and summarised the data for this study using Microsoft SQL server 2012 and Microsoft Excel.

Favourable ethics approval was secured in October 2018 to use the Discover-NOW Research Platform for research purposes for a period of 5 years. The REC reference is 18/WM/0323 and the IRAS project ID is 253449. This research successfully secured local R&D approval to proceed from the NWL Data Research Access Group in August 2019.

### Reporting summary

Further information on research design is available in the Nature Research Reporting Summary linked to this article.

## Supplementary information

Supplementary Information

Reporting Summary
