# Supplementary material for: Risks of poor asthma outcome in 14,405 children and young people in London
Source: NPJ Prim Care Respir Med. 2021 Jan 29;31:3. doi: 10.1038/s41533-020-00215-7 (PMC7846552; doi:10.1038/s41533-020-00215-7)
Supplement: Supplementary file 1 — Supplementary Information [file 41533_2020_215_MOESM1_ESM.pdf]

Risks of poor asthma outcome in 14405 Children and Young People in North West London

Supplementary notes:

The Whole Systems Integrated Care (WSIC) Database developed by a consortium of 8 Clinical Commissioning Groups in North West London (NWL). In addition to research, which is delivered by the Imperial Health Care Partnership (IHCP) with WSIC (1) called Discover-Now, the data is used to generate an up-to-date clinical integrated summary care record stored in a secure dedicated data warehouse. This is accessible to authorised health and social care professionals involved in a patient's treatment, thereby facilitating improved clinical decision making, administration of NHS patient care and is also available for research using a fully de-identified version of the WSIC data in a separate secure data warehouse.

A suite of clinical care dashboards (called RADARs) (2) have been built using the WSIC database and Tableau visualisation software for the combined Health and Social care data across the NW London consortium. These tools support delivery of healthcare by facilitating proactive case finding and are applicable at practice level for clinical needs as well as providing an understanding of population health.

For example the Asthma Radar, part of a suite of chronic disease dashboards, was designed to enable both generalists and asthma trained clinicians to easily identify patients at risk of poor outcome by classifying patients with the most important risk factors into visible 'Red Flag' markers. These are derived from bi-monthly data extraction from primary and secondary care and provides a dynamic 'risk register' for NWL practices. While asthma specialists in primary care will be aware of the risk factors for poor outcome, generalists may not; furthermore it is fairly labour intensive to keep searching individual general practice databases to identify patients in need of care optimisation. The WSIC Asthma Radar available to practices with secure access for patient care: patients' (each on a separate row) risk factors are displayed as 'red flags'

This WSIC asthma Radar has the potential to help clinicians in primary and secondary care irrespective of their level of asthma expertise, to easily identify those patients currently at risk and whose care needs optimising. An example of the asthma Radar (filtered for children in this case) is shown in Supplementary figure 1 where 'red flags' clearly identify patients at risk, who have suffered more than one asthma exacerbation in the previous 12 months, and/or who collected excess short acting beta-agonist bronchodilators (SABAs) and/or insufficient preventer (controller) medication, and/or poor inhaler technique and/or reduced lung function; as well as process measures such as asthma reviews and issue of personal asthma action plans (PAAPs) during the last 12 months. Patients who are flagged at risk by the system may of course be incorrectly labelled with an asthma diagnosis and this is an important reason for reviewing them.

Supplementary Figure 1 : **This is a demonstration illustration of how data is displayed for clinicians and is reproduced with permission from the Head of Deployment Whole Systems Integrated Care (WSIC Dashboards).**

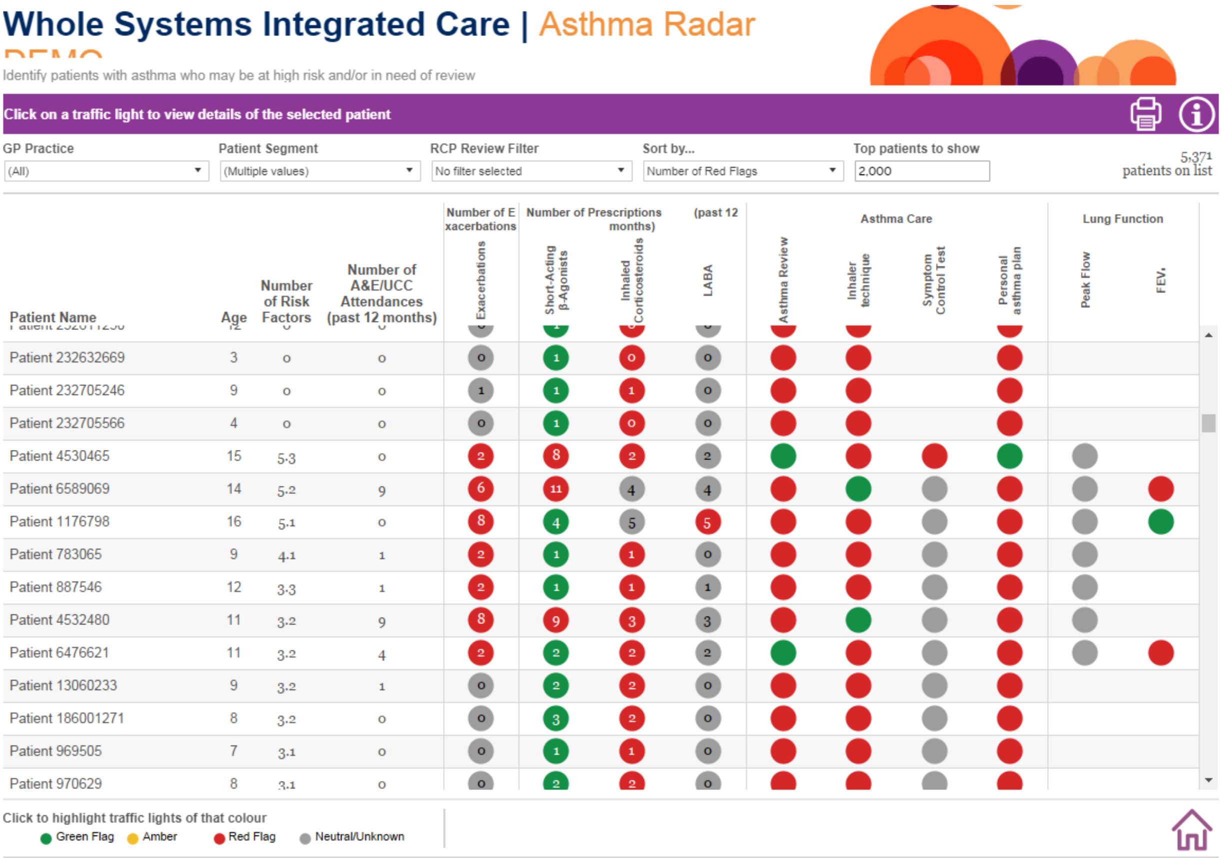

Colum labels : A&E = Accident and Emergency; UCC = General practice urgent care centre; Exacerbations = a) hospital admissions /or b) short courses oral prednisolone/ or c) computer coded asthma attack; LABA = Long Acting Bronchodilator; FEV1 – Forced Expiratory Volume in one second.

Supplementary Figure 2 :

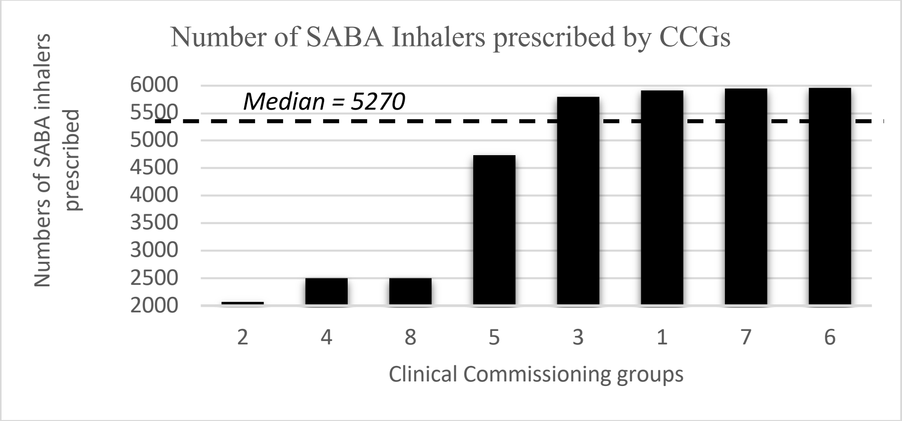

Numbers of short acting beta-agonist bronchodilator (SABA) inhalers prescribed by Clinical Commissioning Group (CCG). Average numbers of SABA inhalers prescribed per year per diagnosed patient per year ranged from 2.29 to 2.73 per CCG

Supplementary Figure 3:

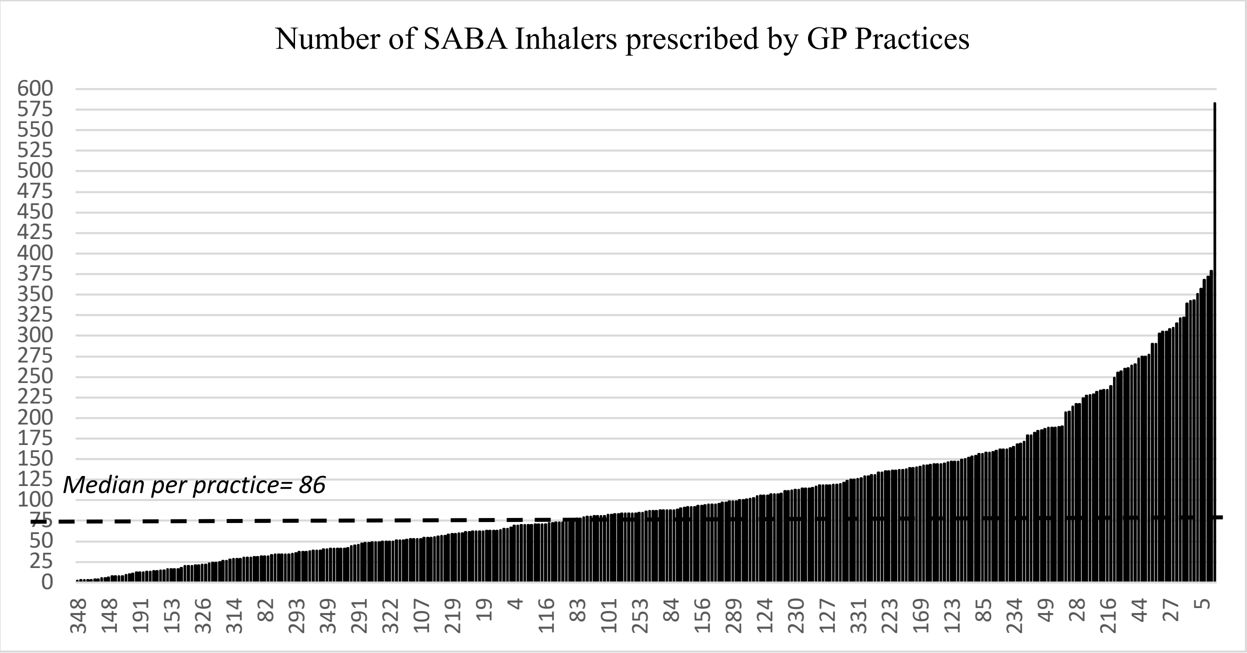

Variation of numbers of SABA Inhalers prescribed by practices during the study year.
Practices prescribed between 0.8 and 10 SABA inhalers per diagnosed patient
(Median 2.43 Q1,Q3: 2.1, 2.85) during the 12 months.

Supplementary Table 1: **Hospital admissions for acute asthma: Numbers and Proportions of Children and Young people (CYP) with diagnosed asthma by Clinical Commissioning Group**

CCG	CYP[a] Diagnosed with Asthma	Number Hospital Admissions for asthma	% Hospital Admissions per CYP with asthma	Cost of admissions per CCG per year	Cost per CYP with asthma per year
1	2176	153	7.03	£187883	£86.34
2	756	45	5.95	£88490	£117.05
3	2337	103	4.41	£129507	£55.42
4	1119	58	5.18	£91280	£81.57
5	1960	95	4.85	£135811	£69.29
6	2458	158	6.43	£206777	£84.12
7	2592	126	4.86	£149634	£57.73
8	1007	59	5.86	£68735	£68.26
Totals	14405	797	5.53	£1058117	£73.45

[a]CYP=Children and Young People <19 years old; CCG=Clinical Commissioning Group

Supplementary Table 2: Frequencies of prescriptions for short acting beta-agonist bronchodilator (SABA) inhalers by age groups in the 12 months from April 2018

**Age group Years**	**Number of SABA[a] prescriptions in 12 months from April 2018**												
	**1**	**2**	**3**	**4**	**5**	**6**	**7**	**8**	**9**	**10**	**11**	**≥12**	**No data[b]**	**Total**
0 - ≤5	210	215	178	111	77	46	29	18	15	7	8	7	112	1033
>5 - ≤14	2452	2240	1416	800	437	285	169	110	89	53	34	58	1275	9418
>14 - <19	1081	792	514	286	187	129	90	58	50	28	20	33	686	3954
**Grand Total**	**3743**	**3247**	**2108**	**1197**	**701**	**460**	**288**	**186**	**154**	**88**	**62**	**98**	**2073**	**14405**

[a]Short Acting Beta-agonist Bronchodilators; [b]Missing data

Supplementary Table 3: : Proportions of Children and young people coded with asthma who were coded with Provision of personal asthma self management plans and who had annual asthma reviews in the eight NW London CCGs

**CCGs**	**Totals of CYP coded with an asthma diagnosis**	**No (%) of total CYP coded as asthma and provided with PAAP**	**No (%) of total CYP coded as asthma and provided with annual asthma reviews**
1	2176	1332 (61)	895 (41)
2	756	424 (56)	445 (59)
3	2337	1869 (80)	1737 (74)
4	1119	425 (38)	693 (62)
5	1960	1182 (60)	827 (42)
6	2458	1352 (55)	1130 (46)
7	2592	1867 (72)	1800 (69)
8	1007	670 (67)	697(69)
**Grand Total**	**14405**	**9121 (63)**	**8224 (57)**

CYP = Children and Young people aged < 19
PAAP = Personal asthma action plans

Bibliography:

1) North West London Collaboration of Clinical Commissioning Groups. *Whole Systems Integrated Care (WSIC) Dashboards and Information Sharing*, https://www.nwlondonccgs.nhs.uk/professionals/whole-systems-integrated-care-wsic-dashboards-and-information-sharing (2020).
2) North West London Collaboration of Clinical Commissioning Groups. *Whole Systems Integrated Care (WSIC) Dashboards: Asthma Radar.*,
.
